# An Unusual Case of Giant Polymicrobial Shoulder Abscess Developing Two Decades After an Insect Bite: The Role of Chronic Intermittent Swelling as a Predisposing Factor

**DOI:** 10.7759/cureus.75816

**Published:** 2024-12-16

**Authors:** Arın Celayir, Muhammed Yusuf Afacan, Baran Sevgil, Burak Ozturk, Onur Yildirim, Huseyin Botanlioglu

**Affiliations:** 1 Department of Orthopaedics and Traumatology, Cerrahpasa Faculty of Medicine, Istanbul University-Cerrahpasa, Istanbul, TUR; 2 Department of Orthopaedics and Traumatology, Kilis Prof. Dr. Alaeddin Yavaşca State Hospital, Kilis, TUR

**Keywords:** caseous abcess, epidermal cyst, insect bite, m. tuberculosis, vacuum assisted closure (vac)

## Abstract

Shoulder abscesses, commonly resulting from bacterial infections, can occasionally present with atypical etiologies and delayed onset. We report a rare case of a massive polymicrobial shoulder abscess developing two decades after an insect bite, emphasizing its clinical presentation, diagnostic approach, and surgical management. A 65-year-old female presented with severe, progressively worsening right shoulder pain, a 20 cm swelling, and purulent discharge persisting for 15 days. Her medical history revealed hypertension, diabetes, and intermittent swelling in the insect-bitten region over the years, which likely predisposed the area to recurrent infections. No evidence of autoimmune disorders, corticosteroid use, or dental abscess was identified. Clinical examination revealed a purulent yellow discharge from the superolateral region of the right shoulder. Ultrasonography and contrast-enhanced MRI confirmed a localized subcutaneous abscess without muscle invasion. The surgical intervention included open irrigation and debridement, revealing dense, purulent, caseous, malodorous material with necrotic debris. Microbiological analysis identified anaerobic streptococci, anaerobic gram-negative bacilli, anaerobic non-spore-forming gram-positive rods, *Corynebacterium* species, and methicillin-sensitive *Staphylococcus aureus* (MSSA), with no evidence of *Mycobacterium tuberculosis*. The pathogens were sensitive to ampicillin-sulbactam. The patient underwent a 10-day intravenous ampicillin-sulbactam regimen, followed by a one-month course of oral amoxicillin-clavulanic acid. The deep wound cavity was managed with vacuum-assisted closure (VAC) therapy for ten sessions, significantly aiding recovery. Complete healing with restored shoulder mobility was achieved. This case highlights the importance of considering unusual etiologies, such as delayed complications from insect bites, in shoulder abscesses. The patient’s history of intermittent swelling in the affected area likely predisposed it to infection. Prompt surgical intervention, appropriate antibiotic therapy, and advanced wound care techniques, including VAC therapy, were pivotal in achieving successful outcomes. Immediate initiation of targeted antibiotics post-sampling effectively managed this polymicrobial infection with a simplified antibiotic regimen.

## Introduction

A shoulder abscess is a localized accumulation of pus in the shoulder region, typically resulting from a bacterial infection. This condition can arise as a complication of skin or soft tissue infections or may originate from deeper structures within the shoulder joint. Common symptoms include severe pain, swelling, redness, and warmth in the affected area. Individuals with weakened immune systems, diabetes, or a recent history of trauma are at a higher risk of developing shoulder abscesses. Prompt medical evaluation is essential for accurate diagnosis and effective treatment, which generally involves abscess drainage and antibiotic therapy. In more severe cases, surgical intervention may be required to address deep-seated abscesses or associated complications [1].

Abscess formation is primarily attributed to bacterial infections, often triggered by breaks in the skin such as wounds, cuts, or insect bites, which provide an entry point for bacteria. Once introduced, bacteria multiply, leading to localized pus formation. Additional risk factors include immunocompromised states, chronic medical conditions like diabetes, and the presence of foreign bodies. Treatment typically involves draining the abscess to remove the accumulated pus, which can be achieved through minor surgical procedures or needle aspiration [2]. Antibiotics are routinely administered to manage the underlying infection and prevent complications. For recurrent or medically complex cases, healthcare providers may implement additional interventions to address underlying causes and reduce the risk of recurrence [3].

This case report describes a rare instance of a giant polymicrobial shoulder abscess that developed 20 years after an insect bite. The presentation, involving purulent and caseous material, highlights an unusual delayed complication, emphasizing the need for a thorough diagnostic approach and tailored treatment strategies in managing atypical abscess presentations.

## Case presentation

The patient, a 65-year-old female, presented to our clinic with increasing pain in the right shoulder, which had started 15 days prior. On physical examination, purulent yellow discharge was observed in the superolateral region of the right shoulder. Informed consent was obtained from the patient prior to any procedures being performed. The patient’s medical history included hypertension and diabetes; however, she did not use medications regularly. Notably, the patient reported an insect bite on the right shoulder approximately 20 years ago, which had caused localized swelling that remained intermittent in size for two decades.

**Figure 1:**
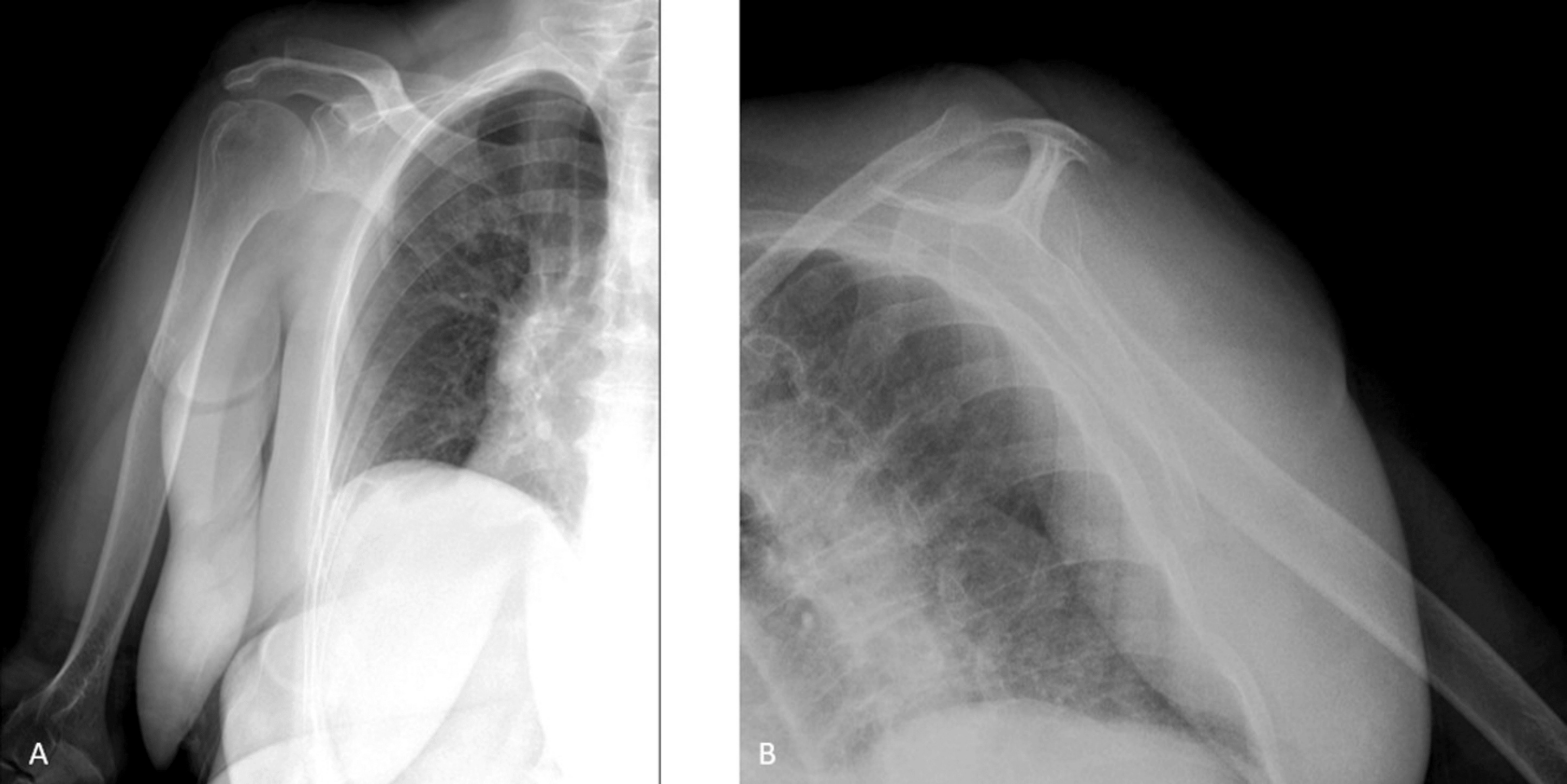
Preoperative X-ray images of the patient. (A) Anteroposterior (AP) view of the shoulder. (B) Scapular Y view of the shoulder.

Initial diagnostic imaging consisted of a direct X-ray followed by an ultrasound, which revealed localized abscess formation in the subcutaneous tissues of the right shoulder (Figure 1).

**Figure 2:**
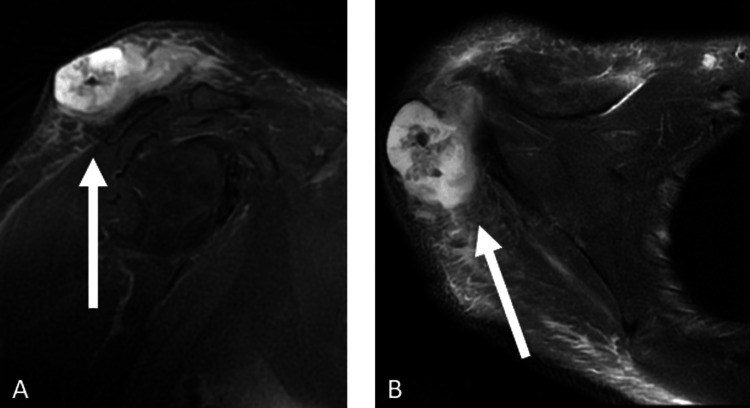
Preoperative magnetic resonance imaging (MRI) with intravenous (IV) contrast of the patient. (A) The pus collection in the sagittal plane is shown. (B) The pus collection in the axial plane is shown. White arrows indicate the abscess.

Subsequently, a contrast-enhanced MRI and the use of ultrasound confirmed the presence of a localized abscess without invasion into the muscle tissues, effectively ruling out malignancy. Radiological evaluation revealed a lesion over the right deltoid muscle with a diameter of 4.5 cm, containing coarse calcifications and lacking significant vascularization. The lesion was reported as either an abscessed lesion or an abscess, considered a differential diagnosis due to the atypical imaging features (Figure 2).

**Figure 3:**
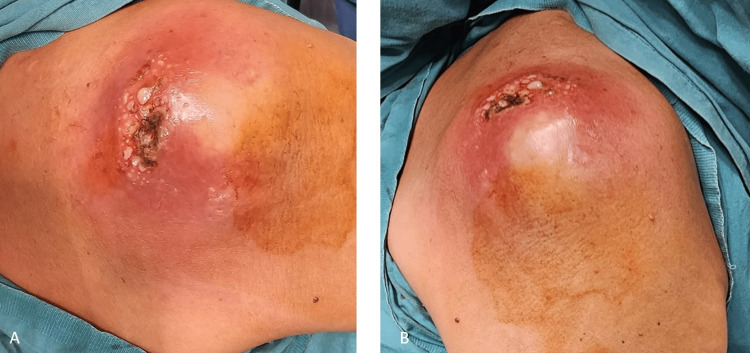
Intraoperative view of the lesion in the right shoulder. (A) Superior perspective. (B) Lateral perspective. The procedure was performed under sterile conditions, and surgical abscess drainage was successfully conducted.

**Figure 4:**
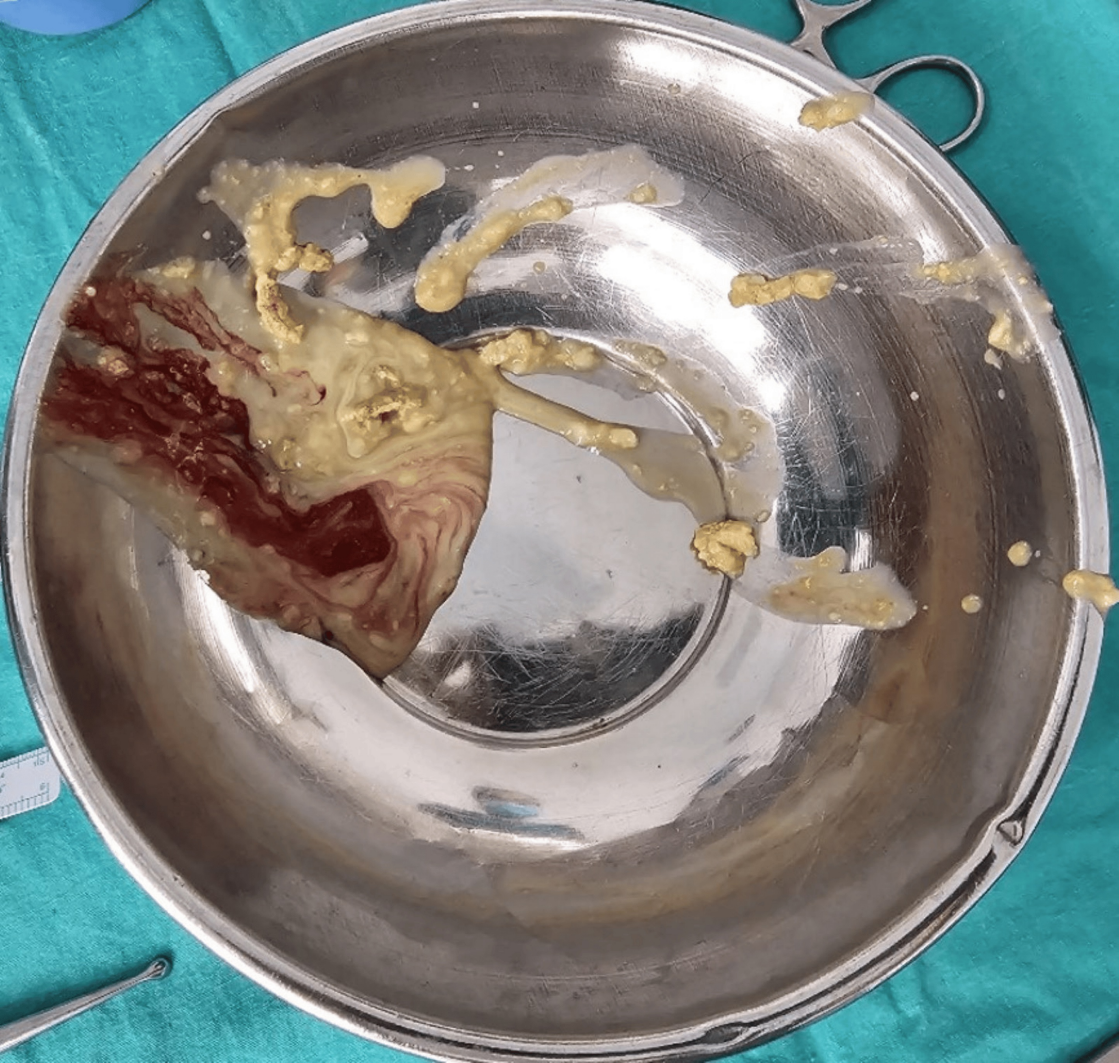
Intraoperative view of the purulent, caseous material removed during surgery, highlighting the dense necrotic debris and the severity of the polymicrobial infection in the giant shoulder abscess.

The patient underwent open irrigation and debridement surgery. Intraoperatively, approximately 20 cc of collected material was observed, characterized as a dense, caseous necrotic structure with a very bad smell (Figures 3-4). The materials were sent for both pathological and microbiological examination.

**Figure 5:**
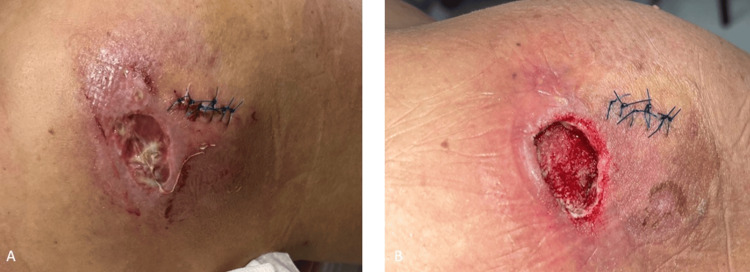
The pouch formed on the patient’s shoulder is shown. (A) The initial state of the pouch. (B) The state after two sessions of VAC therapy.

During the surgery, a large pouch was formed. The pouch was fully filled with purulent, infected, necrotic tissue. We surgically debrided the dead tissue, thoroughly irrigated the area, and applied a vacuum-assisted closure (VAC) system to the opened pouch. The deep wound cavity was managed with vacuum-assisted closure (VAC) therapy for 10 sessions over a month, which significantly contributed to the healing process (Figure 5).

**Figure 6:**
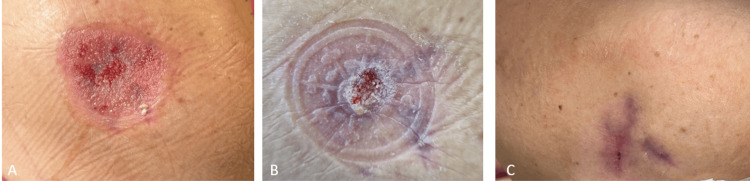
The wound site follow-up images of the patient who underwent serial VAC applications are shown. (A) Clinical image one month after surgery. (B) Clinical image two months after surgery. (C) Clinical image three months after surgery.

The patient fully recovered without any restrictions in shoulder mobility (Figure 6). 

Microbiological analysis results reported the presence of anaerobic streptococci, anaerobic gram-negative rods, non-spore-forming anaerobic gram-positive rods, *Corynebacterium* sp., and methicillin-sensitive *Staphylococcus aureus* (MSSA). No evidence of *Mycobacterium tuberculosis* was found. All identified bacteria were sensitive to ampicillin-sulbactam therapy. The patient was treated with intravenous ampicillin-sulbactam 1g/0.5g (4x2) for 10 days, starting immediately after the surgery, followed by oral amoxicillin-clavulanic acid 1000 mg (2x1) for one month.

## Discussion

The surgical treatment of a shoulder abscess requires a carefully planned approach, beginning with obtaining informed consent. The procedure typically involves thorough exploration and drainage of the abscess through incision and drainage or, in more complex cases, open irrigation and debridement. The primary objectives are to remove purulent material and necrotic tissue to promote optimal healing. In complicated cases, imaging modalities such as ultrasound or MRI may be employed to accurately locate and address deeper structures. Postoperatively, meticulous wound care, including the application of sterile dressings, is essential to prevent secondary infections and facilitate proper healing. The choice of surgical intervention depends on factors such as the size, location, and severity of the abscess, as well as the patient’s overall health.

An insect bite followed by abscess formation is a rare but noteworthy event. Insect bites can introduce bacteria or other pathogens into the skin, triggering an inflammatory response that fosters bacterial growth and abscess development [4]. Prompt medical attention is crucial in such cases to manage the infection and prevent complications. However, in our patient, the history of an insect bite 20 years earlier did not fully explain the acutely developing condition. It is possible that the presence of a long-standing intermittent swelling in the shoulder created a favorable environment for the inoculation of infectious pathogens, leading to the abscess. The rapid emergence of dense, white material within just 15 days raised the suspicion of a hyperacute tuberculosis infection, prompting the initiation of a prolonged antibiotic regimen and close monitoring.

Insect bites, though commonly resulting in minor dermatological manifestations such as erythematous and edematous eruptions, papules, and urticaria, can occasionally lead to severe complications. These include systemic reactions and secondary infections, as arthropod bites serve as potential vectors for bacterial, viral, and protozoal diseases. In the present case, a history of intermittent swelling at the insect bite site likely created a localized inflammatory microenvironment that predisposed the area to infection over time. This rare complication underscores the importance of considering delayed, atypical outcomes in patients presenting with chronic swelling or abscesses at the site of prior insect bites, especially in those with systemic risk factors such as diabetes or immune dysregulation [5-6].

Bacterial infections remain the most common cause of abscess formation, with Staphylococcus aureus, including methicillin-resistant strains (MRSA), being a leading pathogen. Other frequent culprits include *Streptococcus pyogenes* (Group A Streptococcus) and various anaerobic bacteria. These pathogens typically gain entry through skin breaks such as wounds, cuts, or hair follicles. While *Mycobacterium tuberculosis* is more commonly associated with vertebral infections, its involvement in the shoulder joint is rare [7]. A study by Banshelkikar et al. highlighted the successful treatment of a *Mycobacterium tuberculosis *abscess near the scapula with debridement and antibiotic therapy. Inspired by their findings, we promptly performed rapid debridement and initiated aggressive antibiotic therapy to address the infection effectively [8].

Although MRSA is a common pathogen in abscesses, less typical and potentially more dangerous infectious agents, such as *Mycobacterium tuberculosis* and fungal pathogens, must not be overlooked. While tuberculosis may not typically result in rapid caseous abscess formation, the presence of dense, white pus should prompt consideration of this diagnosis. This case underscores the importance of maintaining vigilance for uncommon pathogens in the context of more typical bacterial infections, ensuring a comprehensive approach to diagnosis and treatment [9].

The case of *Mycobacterium* abscess infection by Carver et al. highlights the potential for severe, delayed complications following insect bites, especially in immunocompromised individuals. They also underscored the importance of early diagnosis and tailored treatment in managing atypical post-insect bite infections due to that rare pathogen which we also suspected [10].

## Conclusions

This case underscores the significance of recognizing uncommon etiologies, such as delayed complications arising from insect bites, in the development of shoulder abscesses. The patient’s history of chronic intermittent swelling in the affected region likely created a predisposition for infection. Timely surgical intervention, coupled with targeted antibiotic therapy and advanced wound management techniques like VAC therapy, played a critical role in achieving a successful outcome. Additionally, the immediate initiation of appropriate antibiotics following sample collection proved effective in managing this complex polymicrobial infection with a streamlined antibiotic regimen.
